# Supplementary material for: Calculated Maximal Volume Ventilation (cMVV) as a Marker of Early Respiratory Failure in Amyotrophic Lateral Sclerosis (ALS)
Source: Brain Sci. 2024 Feb 3;14(2):157. doi: 10.3390/brainsci14020157 (PMC10887238; doi:10.3390/brainsci14020157)

Supplementary materials

Supplementary Figure S1. A. Distribution of patients according to FVC%, FEV1% and cMVV(40) values. Only 60 patients (7.9%) resulted to have discordant FVC>80 and FEV1<80, while the 28.8% (N=218) of the whole cohort have normal FVC and MVV(40)<80 (see the blue dashed square). **B. Distribution of patients according to FEV1% and cMVV(40) values.**

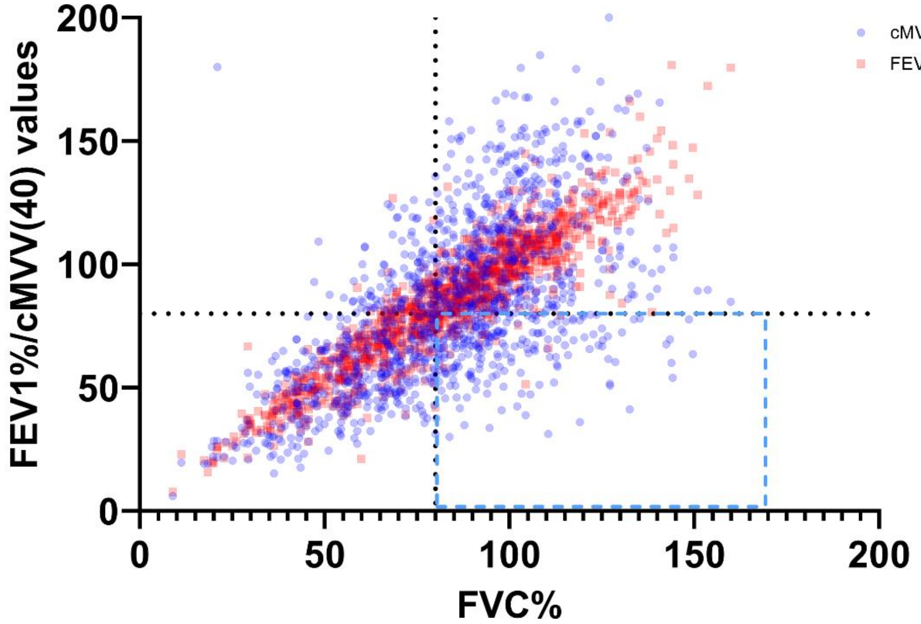

A

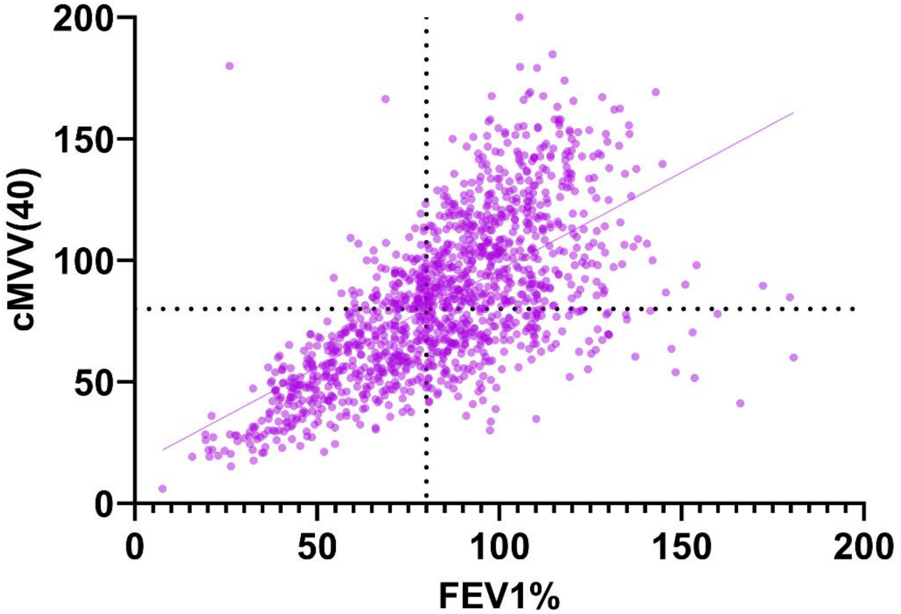

B

Supplementary Table S1. Cox proportional hazard models for cMVV(Dillard) and FEV1%. cMVV(Dillard) showed the same stratification properties as cMVV(40), while FEV1% resulted non-significant after stratification for FVC% <>80.

Multivariate analysis – Overall survival – cMVV(Dillard)

	Adjustments		HR (95% CI)	p
	Age at onset (cont), Sex, Site of onset (B/S), ΔALSFRS (N=576)	cMVV (Dillard) (continuous)	0.988 (0.984-0.992)	**<0.001**
		cMVV (Dillard) (median adj)		
		<85	1	
		≥85	0.718 (0.579-0.892)	**0.003**
		cMVV (40) (quartiles adj)		
		<65	1	
		65-85	0.596 (0.460-0.774)	**<0.001**
		85-110	0.518 (0.398-0.675)	**<0.001**
		≥110	0.443 (0.330-0.594)	**<0.001**
		cMVV (Dillard) (80)		
		<80	1	
		≥80	0.575 (0.460-0.720)	**<0.001**

Stratified analysis – Overall survival – cMVV(Dillard)

FVC ≥80	Age at onset (cont), Sex, Site of onset (B/S), ΔALSFRS (N=576)	cMVV (Dillard) (median adj)		
		<85	1	
		≥85	0.995 (0.742-1.335)	0.975
FVC <80	Age at onset (cont), Sex, Site of onset (B/S), ΔALSFRS (N=576)	cMVV (Dillard) (median adj)		
		<85	1	
		≥85	0.816 (0.554-1.201)	0.302
FVC ≥80	Age at onset (cont), Sex, Site of onset (B/S), ΔALSFRS (N=576)	cMVV (Dillard) (80)		
		<80	1	
		≥80	0.746 (0.576-0.965)	**0.026**
FVC <80	Age at onset (cont), Sex, Site of onset (B/S), ΔALSFRS (N=576)	cMVV (Dillard) (80)		
		<80	1	
		≥80	0.832 (0.568-1.220)	0.347

Stratified analysis – Overall survival – FEV1%

			HR	**p**		HR	**p**
FVC ≥80	Age at onset (cont), Sex, Site of onset (B/S), ΔALSFRS (N=1287)	FEV1% (median adj)			FEV1% (80)		
		<85	1		<80	1	
		≥85	1.105 (0.820-1.490)	0.511	≥80	0.995 (0.730-1.357)	0.976
FVC <80	Age at onset (cont), Sex, Site of onset (B/S), ΔALSFRS (N=1287)	FEV1% (median adj)			FEV1% (80)		
		<85	1		<80	1	
		≥85	1.033 (0.789-1.352)	0.814	≥80	0.976 (0.750-1.270)	0.856

Supplementary Table S2. Cox proportional hazard models according to FVC% and cMVV(40) cut-offs. Patients were subdivided into four categories according to the combination of FVC% values and cMVV(40), using for both the cut-off of 80.

	B	SE	Wald	gl	Sign.	Exp(B)	95,0% CI Lower	Upper
Age at PFT	0.013	0.003	18.009	1	<0.001	1.013	1.007	1.020
Sex	0.274	0.073	14.258	1	<0.001	1.316	1.141	1.517
ΔALSFRS-R	0.170	0.025	47.858	1	<0.001	1.185	1.129	1.244
Site of onset (B/S)	0.181	0.064	8.003	1	0.005	1.198	1.057	1.359
FVC≥80 & cMVV(40)≥80			118.280	3	<0.001			
FVC≥80 & cMVV(40)<80	0.332	0.105	9.910	1	0.002	1.394	1.133	1.714
FVC<80 & cMVV(40)≥80	0.690	0.114	36.419	1	<0.001	1.994	1.594	2.495
FVC<80 & cMVV(40)<80	0.803	0.080	101.873	1	<0.001	2.232	1.910	2.609

Supplementary Figure S2. Kaplan-Meier curves for overall survival according to cMVV(40) cut-offs. Patients were subdivided into three categories according to cMVV(40) cut-offs (≤60; 60-80; ≥80). All pairwise log-rank tests were significant (p<0.001).

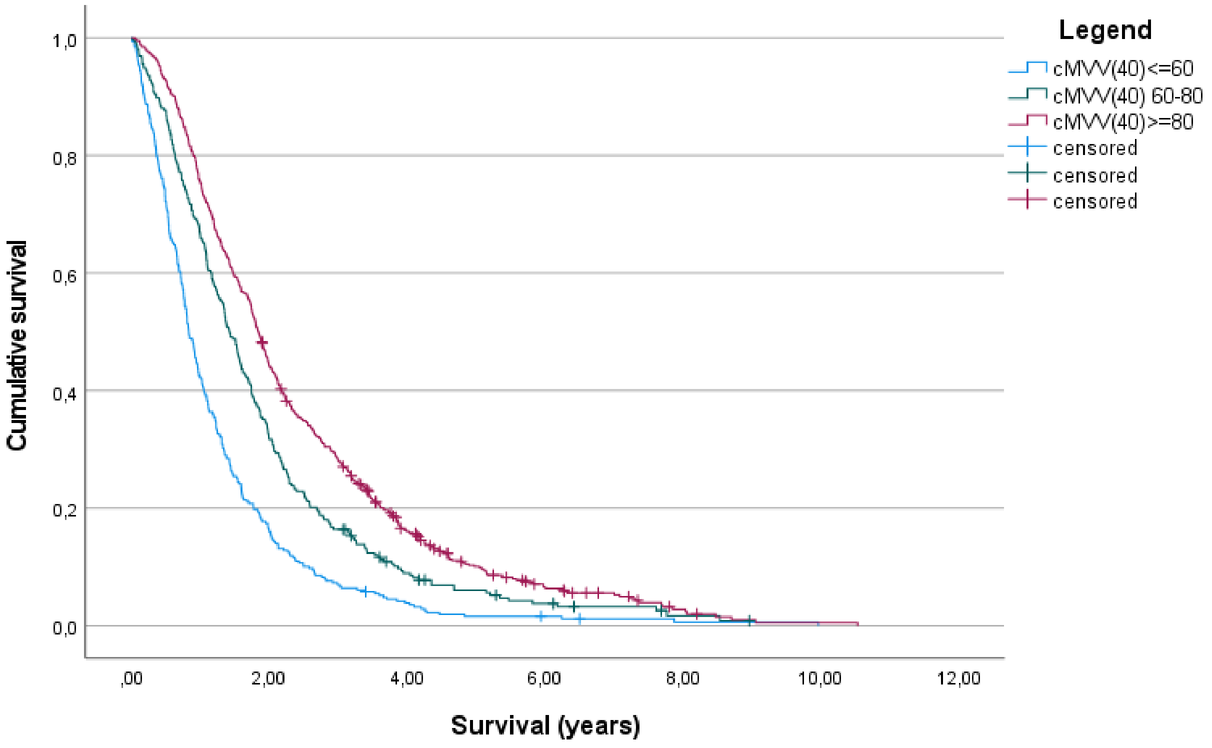

Supplementary Figure S3 – ROC curves for cMVV(40) for survival at 3, 6, and 12 months and NIV start at 6 months. Youden index value for survival at 1 year resulted to be of 77.8 and for NIV at 6 months of 82.6, confirming that values of cMVV(40) around the adjusted median can be considered as valid cut-off for ALS population.

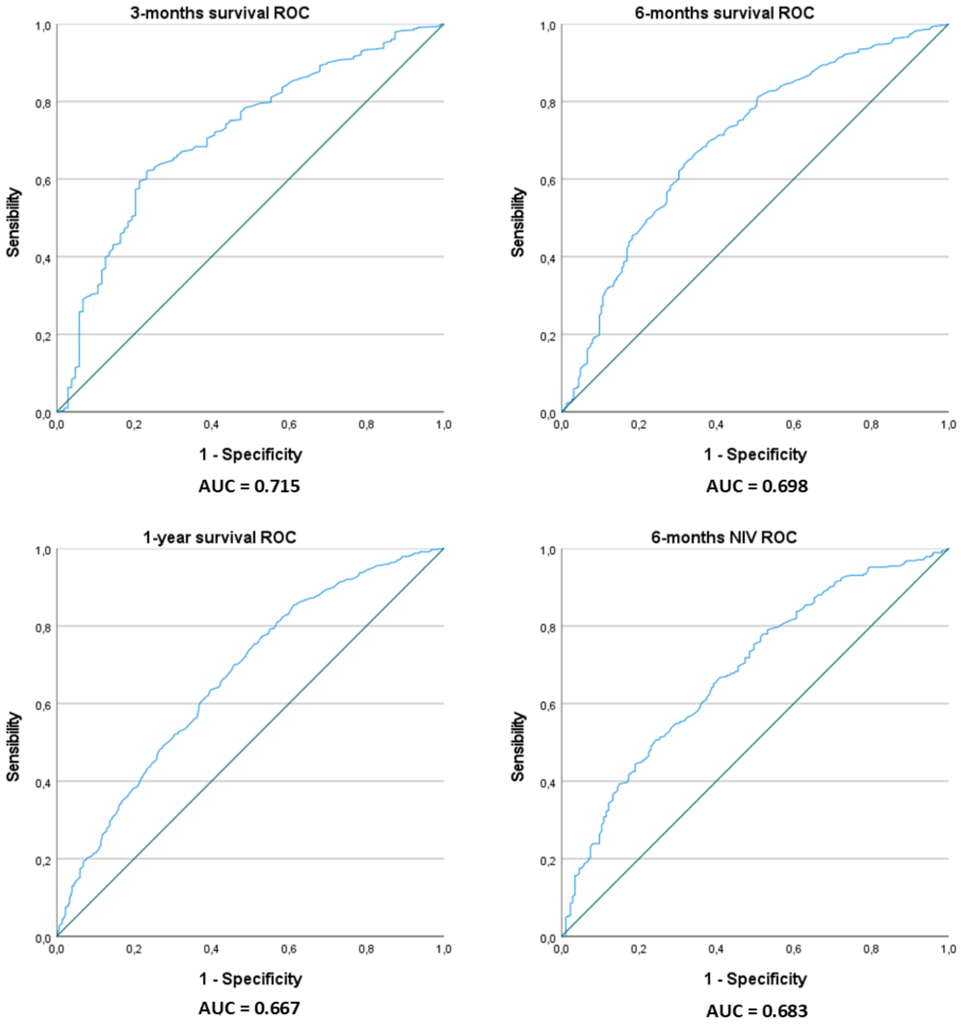

Supplement: Supplementary file 1 [file brainsci-14-00157-s001.zip › brainsci-2759241-supplementary.pdf]
